# Building capacity for African Primary Care Research

**DOI:** 10.4102/phcfm.v6i1.621

**Published:** 2014-02-05

**Authors:** Olayinka O. Ayankogbe

**Affiliations:** 1 Family Medicine Unit, Department of Community Health and Primary Care College of Medicine, University of Lagos, Nigeria

Given the huge burden of disease and the relatively small number of healthcare workers in Africa, the need for strong primary health care and well-trained generalists is high.^1,2^ Primary health care is needed ‘now, more than ever’ if universal health care coverage is to be achieved and the health of African communities improved.^3^ These goals will be dependent to a large extent on the availability of relevant and valid evidence derived from the African primary health care context itself.^4^

Departments of family medicine and primary care throughout the African continent have the potential to make a significant contribution to this body of evidence through primary care research. It is with this in mind that the World Organization of Family Doctors (WONCA), through its Working Party on Research, is attempting to build and promote research capacity in all its regions, including Africa.^5^ Currently, Professor Bob Mash, from Stellenbosch University, and Dr Olayinka Ayankogbe, from the University of Lagos, represent the Africa region on this working party.

The mandate of the WONCA working party is to expand research on general practice, family medicine and primary care and to encourage interested family doctors to participate in research.^5^ Its vision is that research should be a core component of training, scholarship and clinical practice in all nations. All university departments of general practice, family medicine or primary care should therefore support and engage in research and collaborate with primary care practices to address relevant research questions that will benefit patients and communities. The working party also calls on governments to ensure that there are protected funds for primary care research.

In Africa however many departments of family medicine and primary care struggle to perform and supervise research and there is a need to capacitate both faculty members and postgraduate students. As a small step towards building such capacity, the African Journal of Primary Health Care and Family Medicine is launching a series of ten articles on primary care research. The publication of these articles was sponsored by the Stellenbosch University Rural Medical Education Partnership Initiative. The articles will focus on supporting master’s level and residency fellowship research projects, which all departments must currently supervise as part of their Master of Medicine degree and fellowship programmes. The articles will focus on methods commonly used by such postgraduate students: surveys using questionnaires, qualitative research, participatory action research, quality improvement cycles and programme evaluation. The articles will also guide postgraduate students and faculty on how to write a research proposal and the final research report.

We hope that this series will support the development of high quality primary care research that may focus on basic methodological tools, clinical issues, health services and systems or education. We hope to see a blossoming of research in family medicine and primary care, all across the African continent, from Cape Town to Cairo, Lagos to Mogadishu, as well as enhanced capacity amongst academic staff and postgraduate students.

This editorial coincides with the publication of the first article in this African primary care research series, ‘Choosing a topic and developing a proposal’.
